# Clinical evaluation of the effect of 980 nm diode laser and fluoride varnish on dentin hypersensitivity

**DOI:** 10.1590/1807-3107bor-2025.vol39.077

**Published:** 2025-07-07

**Authors:** Sérgio Kiyoshi ISHIKIRIAMA, Bruno Nicollielo MOREIRA, Linda WANG, Juliana Fraga Soares BOMBONATTI, Giovanna Speranza ZABEU, Fabio Antonio Piola RIZZANTE

**Affiliations:** (a) Universidade de São Paulo – USP, Bauru School of Dentistry, Department of Operative Dentistry, Endodontics, and Dental Materials, Bauru, SP, Brazil.; (b) UniSagrado, School of Dentistry, Department of Reconstructive and Rehabilitation Sciences, Bauru, SP, Brazil.; (c) Medical University of South Carolina, James B. Edwards College of Dental Medicine, Department of Oral Rehabilitation, Charleston, SC, USA.

**Keywords:** Dentin Sensitivity, Lasers, Fluorides, Topical

## Abstract

Dentin hypersensitivity (DH) is a common and challenging clinical condition with limited long-lasting treatments. The objective of this study was to evaluate the effectiveness of 980 nm diode laser treatment, associated or not with fluoride varnish, in the treatment of DH. Sixty volunteers were selected and randomly assigned for treatment following three different protocols (1- treatment with 0.8W diode laser; 2- treatment with 0.8 W diode laser over fluoride varnish; or 3- fluoride varnish only). The 0.8 W diode laser was applied in contact with the exposed roots at 10 Hz, for 30 s, with 99.17J/cm^2^ energy density, using a zigzag pattern. DH assessment was performed using the visual analog scale (VAS), prior to treatment, immediately after treatment, and at 7, 30, and 180 days after treatment. The data obtained were subjected to two-way repeated-measures analysis of variance (ANOVA) (p <0.05). The varnish group showed a reduction in DH up to 30 days, whereas the laser and laser + varnish groups showed a reduction in DH up to 180 days ,with no difference between them. The laser and laser + varnish groups were superior to the varnish group after 30 days. The treatment of exposed roots with diode laser alone or associated with fluoride varnish, according to the parameters used in this study, was effective in reducing DH up to six months.

## Introduction

Dentin hypersensitivity (DH) is a common and challenging clinical condition with multifactorial etiology described as an acute painful response to thermal (hot or cold), chemical (acidic fruits, spicy foods, sugar, and salt), mechanical (brushing), and evaporative (air jets) stimuli applied to the clinically exposed dentin, due to the presence of open dentinal tubules.^1-10^ This clinical condition is reported to affect up to 92.1% of the population and to substantially impact patients’ quality of life (QoL), triggering emotional and psychological issues.^6,8,11,12^

Many theories have been created to explain the pain mechanism. The Brannstrom hydrodynamic or fluid movement theory is the most widely accepted one, which states that a fluid movement inside dentinal tubules caused by mechanoreceptor-triggering stimuli in the pulp results in the perception of pain.^5^ Although many treatments have been proposed, most of them based on the physical obliteration of the dentinal tubules and/or desensitization of the nerve endings in the pulp, there is no efficient treatment in the long term. Relapses probably occur because of the multifactorial characteristic of DH and its causal factors: abrasion, erosion, dental malformations, periodontal treatments, and iatrogenesis.^1-4,7,9,10,13^

In addition, although DH is one of the main indicators for root coverage procedures, whether surgical or restorative, there are discussions about its predictability and long-term success, as well as about its higher cost and invasiveness when compared with desensitizing treatments.^10,13,14^

More recently, therapy with high-power lasers has been used to create physical obliteration of open dentinal tubules as a primary effect, and a potential biomodulatory effect as a secondary effect.^8,13,15^ Seeking a better clinical outcome, some authors have studied the association of fluoride varnishes with laser treatment in order to increase the interaction of the laser with the dentin surface and obtain greater and long-lasting tubule obliteration. This association has shown more effective results than just one of the treatments performed alone.^13,16-20^

Despite the promising in vitro results with 980 nm diode laser treatment, showing that laser at 2-4 W reduces diameter, or completely obliterates dentin tubules, there are concerns about the clinical safety and outcomes of such protocols.^17,20,21^ There is a lack of clinical studies associated with a wide variety of parameters ranging from 0.8-3 W in non-contact mode,^15,16,22-25^ while the literature recommends 0.4-1 W power for laser application.^15,26,27^

Moreover, there is scarce information about energy density, and maintenance of the appropriate dosage when laser is applied in non-contact mode poses a clinical challenge because keeping the laser tip at a steady distance may be almost impossible. Therefore, proposing efficient parameters for in-contact application may result in easier treatment, which should be associated with customized treatment times based on the area of exposed dentin.

Considering there is a wide variety of protocols and lasers used in the clinical setting and lack of adequate information about the clinical use of 980 nm diode laser in the treatment of DH,^10,13,15^ this study was proposed as an extension of an in vitro study that showed a significant reduction in dentin permeability after treatment with 980 nm laser associated or not with fluoride varnish application.^13^ Therefore, this study aimed to clinically evaluate the efficacy of 980 nm diode laser therapy, associated or not with the application of fluoride varnish, in the treatment of DH. The null hypotheses tested were: a) There would be no difference in the reduction of DH at the different evaluation times; and b) There would be no difference in the reduction of DH considering the different treatments.

## Methods

### Experimental design

This parallel interventional triple-blind (patients, operators responsible for post-treatment evaluation, and statistician) randomized study assessed the treatment technique at three levels (fluoride varnish, laser therapy, and laser therapy + fluoride varnish). The response variable was the pain intensity after evaporative stimulus measured through a visual analog scale (VAS) assessed before treatment, immediately after treatment, and at 1 week, 1 month, and 6 months after treatment.

### Sample size calculation

A minimum of 13 subjects per group was estimated based on an expected standard deviation of 1.4^28^ and a minimum detectable difference in means of 2, using a test power of 90% and alpha level of 0.05.

### Clinical trial registration and research ethics committee

This clinical trial was registered at ReBEC under the number RBR-7q7gyd, UTN code U1111-1254-9691 (REBEC (ensaiosclinicos.gov.br). This study project was submitted to and approved by the Research Ethics Committee of Bauru School of Dentistry - University of São Paulo (FOB/USP) under CAAE: 49808715.5.0000.5417. This study was conducted in accordance with the 2013 revised version of the Declaration of Helsinki .

### Selection of volunteers

**Figure 1:**
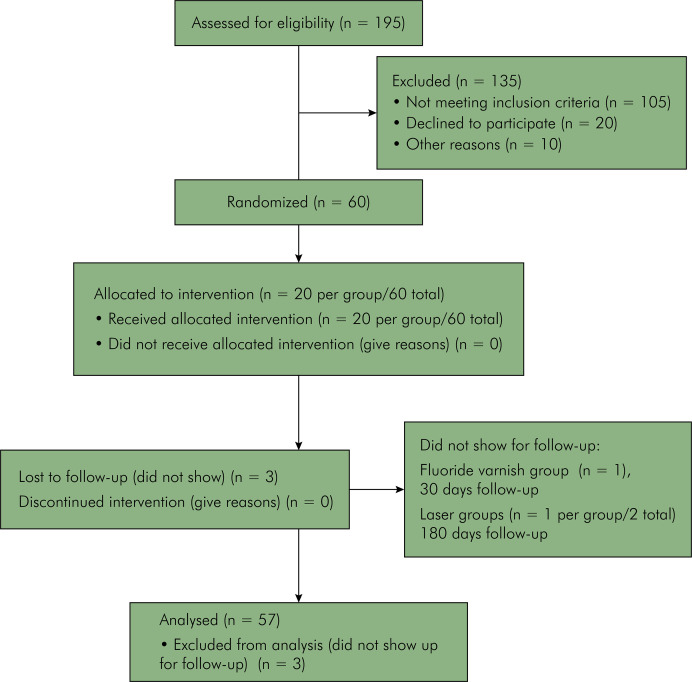
Patients flowchart.

After registration and approval by the local ethics committee, a total of 60 out 195 volunteers were selected by a previously calibrated operator based on inclusion and exclusion criteria (Table 1). The first posted date for this study was July 2020. All patients were selected and treated between January and November 2021 (not including the follow-up period) (Figure 1).

**Table 1:** Inclusion and exclusion criteria.

Inclusion	Exclusion
18–50 years old	Be under psychological counseling
At least 1 tooth with an exposed root with dentin sensitivity greater than 3 on the Visual Analogue Scale (VAS), measured after evaporative stimulus (air jet at 1 cm from the root surface for 3 seconds);	Chronic use of medications with analgesic, anti-inflammatory or drugs with central effect (anxiolytics and antidepressants);
Miller’s class I or II gingival recession^25^ only on the buccal surface, with an extension between 2 and 5 mm.	Use of desensitizing agents (at home) in the last 3 months or in the office (6 months), including fluoridated mouthwashes
No active caries lesions in any tooth	Pregnancy
No active periodontal disease	Allergy to any of the treatment components
Assessed teeth not an abutment for fixed or removable partial dentures	Being under orthodontic treatment
Presence of deep, non-carious cervical lesions that need root restoration (≥1mm)	Presence of a furcation lesion in the selected teeth

### Clinical evaluation and treatment

All volunteers were initially evaluated and treated by the same previously calibrated operator (B.N.M) at the Clinical Research Center of the University. Prior to treatment, all patients underwent dental prophylaxis with rubber cup and fluoride-free toothpaste.

To identify the baseline dentinal sensitivity of each patient, the most sensitive tooth was identified and isolated using cotton rolls, allowing the evaporative stimulus (air jet) to be applied on the clinically exposed root.^16^

**Figure 2:**
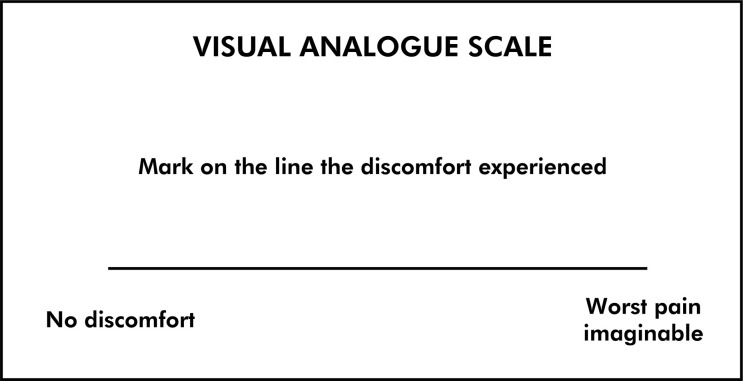
Visual analog scale.

The air jet was applied using an air syringe positioned perpendicularly to the area of dentin exposure, at a distance of 1 cm, for 3 s. Immediately after removing the stimulus, patients reported the pain intensity by marking a trace on a 10-cm line where the left end represented absolutely no discomfort and the right end represented the worst pain imaginable (visual analog scale - VAS) (Figure 2). The line was measured from the left end to the trace marked to obtain a VAS value.

**Figure 3:**
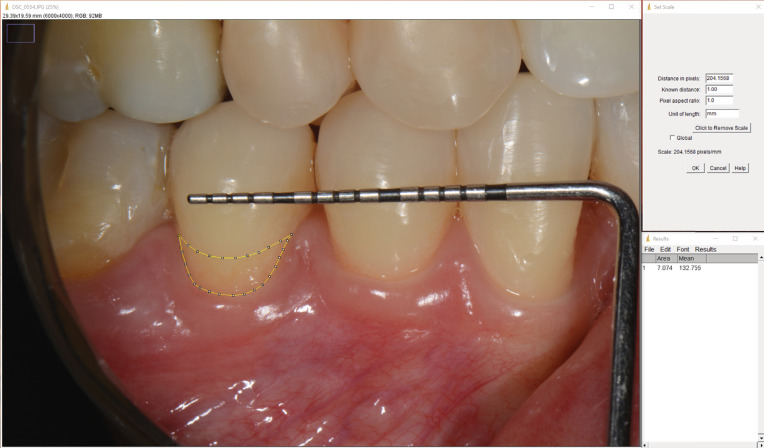
Calculation of exposed root area based on clinical picture.

In addition, all roots were photographed with a digital camera (Nikon 5200, ISO 100, 1/160, Nikon micro 105mm f / 29, Twin Flash TTL, Tokyo, Japan), positioned perpendicularly to the area of root exposure, with a periodontal probe positioned over the coronal portion of the tooth. The captured images were analyzed using the ImageJ software (National Institute of Health, Bethesda, USA), which allowed calculating the exposed root area. The periodontal probe was used to calibrate the measurements within the software program (Figure 3).

### Sample randomization

The 60 patients were randomly distributed (B.N.M) into three study groups according to their pain intensity when exposed to the evaporative stimulus, using Microsoft Excel in a way that ensured all groups had similar initial reported values of sensitivity (Table 2).

**Table 2:** Mean and standard deviation of pain intensity values (VAS) recorded by patients after evaporative stimulus (n = 19 per group).

Variable	Age	baseline	Immediate	7 days	30 days	180 days
varnish	34.5 ± 9.7	5.92 ± 2.27Aa	3.24 ± 2.69Ab	3.74 ± 2.58Bb	4.18 ± 2.72Bbc	5.22 ± 2.27Bac
laser	32.4 ± 8.1	6.03 ± 2.00Aa	2.26 ± 1.66Ab	1.92 ± 1.65ABb	1.86 ± 1.76Ab	1.92 ± 1.63Ab
varnish + laser	33.4 ± 9	5.96 ± 1.88Aa	1.32 ± 1.08Ab	1.48 ± 1.14Ab	1.45 ± 1.00Ab	1.42 ± 1.05Ab

Uppercase letters mean statistically significant difference between rows in the same column (different treatments at the same timepoint) Lowercase letters mean statistically significant difference between columns in the same row (same treatment at different timepoints).

### Treatment protocols

#### Fluoride varnish

This study used a 5% colorless sodium fluoride varnish with 22,600 ppm of fluoride (Clinpro™ White Varnish; 3M, Campinas, Brazil), applied with a microbrush over the total area of clinically exposed dentin. The varnish was applied in a single layer on the dry dentin surface under isolation with cotton rolls. To simulate laser treatment, the laser tip was positioned over the area of exposed dentin for 30 s without touching the fluoride varnish. Five minutes after application, the patient was instructed to moisten the region using the tongue to activate the product according to the manufacturer’s instructions and to avoid drinking fluids for 1 h.

#### Diode laser

**Figure 4:**
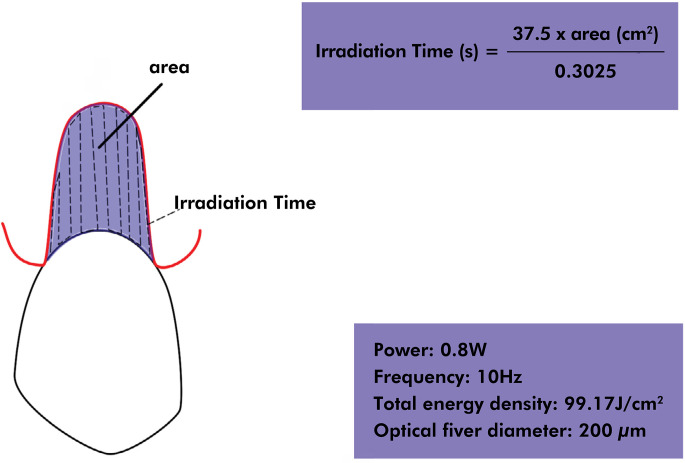
Schematics of laser irradiation pattern.

This study used a 980 nm diode laser (DClase, DC International LLC, Wellington, USA), capable of generating a power between 0.5 W and 7 W. The laser was applied over the exposed root surface under isolation with cotton rolls through a 200-μm diameter optical fiber in contact mode, perpendicularly to the root dentin, with a standardized sweeping movement in a zigzag pattern such that the laser passed only once at each point on the tooth surface (Figure 4). The laser tip was cleaved after each session to ensure standardization.

The laser parameters were 0.8 W power, 10 Hz frequency, and total energy density of 99.17J/cm^2^. These parameters were referenced from a previous laboratory study^13^ from which the best dentin sealing outcomes were obtained at an energy density of 99.17 J/cm^2^, irradiating 0.8 W and 1 W over a surface of 0.3025 cm^2^ for 30 s. To maintain the same energy density, the following formula was used to determine the irradiation time regarding the different areas of the exposed roots (previously calculated using the clinical photos and ImageJ software):


 Irradiation time =(37.5× area )/0.3025

#### Diode laser + fluoride varnish

The colorless fluoride varnish was applied as described above, immediately followed by laser application with the optical fiber in contact with the varnish and with the root dentin. At the end of each application, the optical fiber was cleaved because of its saturation with varnish.

For all three groups, the evaporative stimulus was applied by two blinded operators (S.K.I and L.W.) on the treated tooth isolated with cotton rolls 15 min after treatment. The evaporative stimulus was repeated at 7, 30, and 180 days after treatment.

Three patients did not show up for the scheduled follow-up visits and were not considered in the results (one in the fluoride varnish group at 30 days and one in each of the other two groups at 180 days) (Figure 1).

## Statistical analysis

The results obtained by the VAS in all evaluation time periods were subjected to two-way repeated-measures analysis of variance (ANOVA) considering treatment and time as study factors, followed by Tukey’s HSD post-hoc test. All statistical analyses were performed using α = 0.05.

## Results

All groups presented similar age and sex distribution (five males and 15 females in each group), as well as similar initial values of sensitivity to the evaporative stimulus (Table 2).

The results obtained by the VAS at the different timepoints are described in Table 2. All treatments resulted in similar VAS scores immediately after treatment. Nevertheless, from 7 days to 180 days, the groups treated with laser exhibited lower sensitivity than the group treated with varnish only.

When considering the effectiveness of each treatment, all groups promoted reduction in DH. Nevertheless, the reduction in the VAS scores was lower for the group treated with varnish only. Moreover, after 180 days, the VAS scores for the group treated with varnish only were similar to the baseline scores. The laser groups associated or not with fluoride varnish application showed reduction of VAS scores immediately after treatment and remained stable at all evaluated timepoints.

In the varnish group, VAS ≤ 1 was observed in five patients after 7 days, in three patients after 30 days, and in no patients after 180 days. In the laser group, seven patients showed VAS ≤ 1 after 7, 30, and 180 days. In the group treated with varnish + laser, nine patients showed VAS ≤ 1 after 7 days as compared to eight patients after 30 and 180 days.

## Discussion

Dentin hypersensitivity is a common and challenging oral condition described as acute dental pain associated with thermal, chemical, mechanical and/or evaporative stimuli due to the presence of open dentinal tubules.^1-9,22^ It is noteworthy that large portions of exposed dentin may not respond to stimuli, while microscopic exposures can be extremely sensitive.^9^ In the present study, only patients with exposed roots presenting sensitivity 3 or higher according to the VAS, without the presence of any other potential pain-causing pathological agents, were selected.

In recent years, several studies have assessed the use of products containing sodium fluoride (NaF) and laser treatment with promising short-term outcomes,^10,12,16,22,23,25,29^ which is in agreement with the findings of this study, showing effective reduction of DH up to 6 months.

Regarding the NaF varnish, the literature has reported limited success in the control of DH, with recurrent symptoms after 3 months or less,^1^ thereby corroborating the findings of this study. The fluoride varnish alone may have inferior stability due to the formation of calcium fluoride (CaF_2_), which is loosely attached to the tooth’s surface and may be removed due to the intraoral challenges promoted by mechanical (toothbrushing, mastication, and occlusion) and acid challenges (feeding, dilution in saliva, etc).^7^

The 980 nm laser is relatively new, with a wavelength close to the absorption peak of hydroxyapatite, causing fewer thermal effects on the tooth pulp and, therefore, making it safer for pulp tissues when compared with other lasers at lower wavelengths.^21,30,31^

A recent systematic review^32^ has reported only four studies using 940 to 980 nm diode laser with settings ranging from 0.3W to 3W for 10 to 120 s, with positive results for the control of DH, especially when associated with another desensitizing agent up to 1 month^15,24,25^ or 3 months.^16^ All reported studies applied laser in non-contact mode, with different pulse parameters, except for Pourshahidi et al.,^15^ who utilized laser in contact mode.

Other clinical studies have demonstrated better outcomes using laser or laser + desensitizing agents when compared to desensitizing agents only.^22,23,29,33^ Nevertheless, there is a lack of standardization regarding dosage. To the best of the authors’ knowledge, this is the first clinical study proposing customized treatment parameters based on the area of dentin exposure, using laser in contact mode. The authors believe laser application in contact mode is clinically easier, especially considering the maintenance of energy density and dosage during treatment, which would be extremely challenging in non-contact mode.

According to the literature, the immediate effect of diode laser application relies on two mechanisms: the laser effects on activation of the sodium-potassium pump within the cell membrane of non-myelinated fibers (C) of the dental pulp, thus increasing the pain threshold;^34^ and the reduction in the diameter of dentinal tubules.^21^ The long-term effects of laser treatment are attributed to the activation of metabolic processes resulting in production of sclerotic dentin and tertiary dentin.^31,35,36^

Considering the association of laser and fluoride varnish, it has been proposed that the laser energy promotes higher NaF varnish adhesion to the dentinal tubules.^13,23^ In an in vitro study, the Ca to P ratio was reported to increase after laser treatment associated with fluoride varnish application, which was attributed to the laser-induced calcium fluoride (CaF_2_) adsorption onto the tooth surface, preventing mineral losses.^13^

One could question the benefits of a treatment that would eventually not be as stable when compared to a potential definitive treatment such as surgery or restorations for root coverage.^8^ Nevertheless, laser treatment of exposed roots is a very conservative, more comfortable, and easier-to-conduct method, even if it needs to be performed more often.

Another pertinent question would be about the safety of the chosen parameters. The researchers previously performed a pilot study about the increase in intrapulpal chamber temperature in extracted bovine teeth with 0.5 mm remaining buccal dentin thickness and observed that irradiation with 0.5 W and 0.8 W, following the same parameters as in this clinical study, resulted in a temperature increase lower than 2.5^o^C while irradiation with 1 W laser resulted in an average increase of 3^o^C. During the pilot study, the authors simulated the worst possible conditions, with only 0.5 mm of dentin between the thermal probe and the laser tip. Moreover, no pulpal circulation was simulated, and a thermal paste was applied to allow for optimal contact between the remaining dentin and the thermal probe. Another in vitro study from our group^13^ showed 0.8 W and 1 W laser treatment had similar dentin hydraulic conductance results. Therefore, the authors chose 0.8 W because it seems to result in appropriate outcomes and minimize risks. This choice is supported by a recent study suggesting 0.8 W for 10 s has the best efficiency versus safety parameters for 980 nm laser.^30^

One of the main limitations of this study was the relatively small sample size, which did not allow for evaluation of the influence of patient demographics in the reported outcomes. Moreover, there was no control group to evaluate possible placebo effects. Nevertheless, the fact that the varnish-only group, despite having a “simulated laser application” as part of the triple-blind protocol, showed recurrence of DH similar to baseline demonstrates that the laser-treated groups had a longer-lasting clinical improvement. It is also noteworthy that although negative control groups are well accepted for short-term clinical trials, to leave a patient without treatment for long periods of time may create ethical issues.

In addition, even with extensive training and calibration of the operator, it is impossible to absolutely standardize the irradiation pattern as in a laboratory study. Therefore, this study simulates a clinical scenario in which it is impossible to guarantee all root surface areas were equally irradiated. Moreover, transferring the pain intensity to a paper scale (VAS) introduces subjectivity, and alternative methods to evaluate DH, such as laser Doppler flowmetry, are then suggested for future studies.^37^

It is also noteworthy that the present study used a 980 nm diode laser, and lasers operating at different wavelengths should promote different results, even with similar energy densities.^38^ Generally, diode lasers are the most affordable and commonly available in dental offices due to their portability and versatility, and they are used for tissue incision, coagulation, and hemostasis combined with the benefits of biostimulation and activation of fibroblasts and osteoblasts.^39^

Based on the present results, it was possible to observe that the treatment of DH with diode laser using the suggested protocol, associated or not with fluoride varnish, was effective both immediately and for up to 6 months.

## Conclusion

Treatment with fluoride varnish was effective in reducing DH for up to 1 month. Treatments with diode laser associated or not with fluoride varnish promoted higher reduction in DH after 7 days, remaining stable up to 180 days.

## Data Availability

Data is available on demand from the reviewers.

## References

[1] Wang L, Magalhães AC, Francisconi-Dos-Rios LF, Calabria MP, Araújo D, Buzalaf M (2016). Treatment of dentin hypersensitivity using nano-hydroxyapatite pastes: a randomized three-month clinical trial. Oper Dent.

[2] Machado A, Sakae L, Niemeyer SH, Carvalho TS, Amaechi B, Scaramucci T (2020). Anti-erosive effect of rinsing before or after toothbrushing with a fluoride/stannous ions solution: an in situ investigation: application order of fluoride/tin products for erosive tooth wear. J Dent.

[3] Galvão AD, Zeola LF, Moura GF, Teixeira DN, Gonzaga RC, Silva GR (2019). A long-term evaluation of experimental potassium oxalate concentrations on dentin hypersensitivity reduction: a triple-blind randomized clinical trial. J Dent.

[4] Patel VR, Shettar L, Thakur S, Gillam D, Kamala DN (2019). A randomised clinical trial on the efficacy of 5% fluorocalcium phosphosilicate-containing novel bioactive glass toothpaste. J Oral Rehabil.

[5] Brännström M (1966). The hydrodynamics of the dental tubule and pulp fluid: its significance in relation to dentinal sensitivity. Annu Meet Am Inst Oral Biol.

[6] Favaro Zeola L, Soares PV, Cunha-Cruz J (2019). Prevalence of dentin hypersensitivity: systematic review and meta-analysis. J Dent.

[7] Francisconi-Dos-Rios LF, Dantas LM, Calabria MP, Pereira JC, Mosquim V, Wang L (2021). Obliterating potential of active products for dentin hypersensitivity treatment under an erosive challenge. J Dent.

[8] Liu XX, Tenenbaum HC, Wilder RS, Quock R, Hewlett ER, Ren YF (2020). Pathogenesis, diagnosis and management of dentin hypersensitivity: an evidence-based overview for dental practitioners. BMC Oral Health.

[9] Moraschini V, Costa LS, Santos GO (2018). Effectiveness for dentin hypersensitivity treatment of non-carious cervical lesions: a meta-analysis. Clin Oral Investig.

[10] Marto CM, Paula AB, Nunes T, Pimenta M, Abrantes AM, Pires AS (2019). Evaluation of the efficacy of dentin hypersensitivity treatments: a systematic review and follow-up analysis. J Oral Rehabil.

[11] Douglas-de-Oliveira DW, Vitor GP, Silveira JO, Martins CC, Costa FO, Cota LO (2018). Effect of dentin hypersensitivity treatment on oral health related quality of life: a systematic review and meta-analysis. J Dent.

[12] Pantuzzo ES, Cunha FA, Abreu LG, Esteves Lima RP (2020). Effectiveness of diode laser and fluoride on dentin hypersensitivity treatment: A randomized single-blinded clinical trial. J Indian Soc Periodontol.

[13] Rizzante FA, Maenosono RM, Duarte MA, Furuse AY, Palma-Dibb RG, Ishikiriama SK (2016). In vitro evaluation of dentin hydraulic conductance after 980 nm diode laser irradiation. J Periodontol.

[14] Chambrone L, Tatakis DN (2015). Periodontal soft tissue root coverage procedures: a systematic review from the AAP Regeneration Workshop. J Periodontol.

[15] Pourshahidi S, Ebrahimi H, Mansourian A, Mousavi Y, Kharazifard M (2019). Comparison of Er,Cr:YSGG and diode laser effects on dentin hypersensitivity: a split-mouth randomized clinical trial. Clin Oral Investig.

[16] Umberto R, Claudia R, Gaspare P, Gianluca T, Alessandro V (2012). Treatment of dentine hypersensitivity by diode laser: a clinical study. Int J Dent.

[17] Liu Y, Gao J, Gao Y, Xu S, Zhan X, Wu B (2013). In vitro study of dentin hypersensitivity treated by 980-nm diode laser. J Lasers Med Sci.

[18] Doppalapudi H, Kancharla AK, Nandigam AR, Sheema Tasneem M, Gummaluri SS, Dey S (2023). Comparative evaluation of diode laser alone and in combination with desensitizing toothpaste in occlusion of dentinal tubules: a SEM study. J Oral Biol Craniofac Res.

[19] Reddy GV, Akula S, Malgikar S, Babu PR, Reddy GJ, Josephin JJ (2017). Comparative scanning electron microscope analysis of diode laser and desensitizing toothpastes for evaluation of efficacy of dentinal tubular occlusion. J Indian Soc Periodontol.

[20] Solati M, Fekrazad R, Vahdatinia F, Farmany A, Farhadian M, Hakimiha N (2022). Dentinal tubule blockage using nanobioglass in the presence of diode (980 nm) and Nd:YAG lasers: an in vitro study. Clin Oral Investig.

[21] Umana M, Heysselaer D, Tielemans M, Compere P, Zeinoun T, Nammour S (2013). Dentinal tubules sealing by means of diode lasers (810 and 980 nm): a preliminary in vitro study. Photomed Laser Surg.

[22] Raichur PS, Setty SB, Thakur SL (2013). Comparative evaluation of diode laser, stannous fluoride gel, and potassium nitrate gel in the treatment of dentinal hypersensitivity. Gen Dent.

[23] Suri I, Singh P, Shakir QJ, Shetty A, Bapat R, Thakur R (2016). A comparative evaluation to assess the efficacy of 5% sodium fluoride varnish and diode laser and their combined application in the treatment of dentin hypersensitivity. J Indian Soc Periodontol.

[24] Tabibzadeh Z, Fekrazad R, Esmaeelnejad A, Shadkar MM, Khalili Sadrabad Z, Ghojazadeh M (2018). Effect of combined application of high- and low-intensity lasers on dentin hypersensitivity: a randomized clinical trial. J Dent Res Dent Clin Dent Prospect.

[25] Raut CP, Sethi KS, Kohale B, Mamajiwala A, Warang A (2018). Evaluation of diode laser and stannous fluoride in the treatment of root sensitivity after access flap surgery: randomized controlled clinical trial. J Indian Soc Periodontol.

[26] Brignardello-Petersen R (2017). Cyanoacrylate and laser treatment result in small improvement in oral health-related quality of life for patients with dentin hypersensitivity. J Am Dent Assoc.

[27] Sgreccia PC, Barbosa RE, Damé-Teixeira N, Garcia FC (2020). Low-power laser and potassium oxalate gel in the treatment of cervical dentin hypersensitivity-a randomized clinical trial. Clin Oral Investig.

[28] Zhu M, Li J, Chen B, Mei L, Yao L, Tian J (2015). The effect of calcium sodium phosphosilicate on dentin hypersensitivity: a systematic review and meta-analysis. PLoS One.

[29] Yilmaz HG, Kurtulmus-Yilmaz S, Cengiz E (2011). Long-term effect of diode laser irradiation compared to sodium fluoride varnish in the treatment of dentine hypersensitivity in periodontal maintenance patients: a randomized controlled clinical study. Photomed Laser Surg.

[30] Meng Y, Huang F, Wang S, Huang X, Lu Y, Li Y (2023). Evaluation of dentinal tubule occlusion and pulp tissue response after using 980-nm diode laser for dentin hypersensitivity treatment. Clin Oral Investig.

[31] Naghsh N, Kachuie M, Bijari M, Birang R (2022). Evaluation of the effects of 980 and 810-nm high-level diode lasers in treating dentin hypersensitivity: A double-blinded randomized clinical trial. Dent Res J (Isfahan).

[32] Abdelkarim-Elafifi H, Parada-Avendaño I, Arnabat-Domínguez J (2022). Parameters used with diode lasers (808-980 nm) in Dentin hypersensitivity management: a systematic review. J Lasers Med Sci.

[33] Femiano F, Femiano R, Lanza A, Festa MV, Rullo R, Perillo L (2013). Efficacy of diode laser in association to sodium fluoride vs Gluma desensitizer on treatment of cervical dentin hypersensitivity: a double blind controlled trial. Am J Dent.

[34] Bagis S, Comelekoglu U, Sahin G, Buyukakilli B, Erdogan C, Kanik A (2002). Acute electrophysiologic effect of pulsed gallium-arsenide low energy laser irradiation on configuration of compound nerve action potential and nerve excitability. Lasers Surg Med.

[35] Dilsiz A, Aydin T, Emrem G (2010). Effects of the combined desensitizing dentifrice and diode laser therapy in the treatment of desensitization of teeth with gingival recession. Photomed Laser Surg.

[36] Carvalho TS, Lussi A (2017). Age-related morphological, histological and functional changes in teeth. J Oral Rehabil.

[37] Miron M, Lungeanu D, Ciora E, Ogodescu E, Todea C (2020). Using laser-doppler flowmetry to evaluate the therapeutic response in dentin hypersensitivity. Int J Environ Res Public Health.

[38] Osmari D, Fraga S, Ferreira AC, Eduardo CP, Marquezan M, Silveira BL (2018). In-office Treatments for dentin hypersensitivity: a randomized split-mouth clinical trial. Oral Health Prev Dent.

[39] Azma E, Safavi N (2013). Diode laser application in soft tissue oral surgery. J Lasers Med Sci.

